# *Ulva prolifera* Extract Alleviates Intestinal Oxidative Stress *via* Nrf2 Signaling in Weaned Piglets Challenged With Hydrogen Peroxide

**DOI:** 10.3389/fimmu.2020.599735

**Published:** 2020-10-30

**Authors:** Yanzhong Feng, Zhimin An, Heshu Chen, Xinmiao He, Wentao Wang, Xiang Li, Haifeng Zhang, Fenglan Li, Di Liu

**Affiliations:** ^1^ Institute of Animal Husbandry, Heilongjiang Academy of Agricultural Sciences, Harbin, China; ^2^ Information Department for Epidemic Prevention, Heilongjiang Provincial Animal Epidemic Prevention and Control Center, Harbin, China; ^3^ Hunan Co-Innovation Center of Animal Production Safety, CICAPS, College of Animal Science and Technology, Hunan Agricultural University, Changsha, China; ^4^ College of Life Sciences, Northeast Agricultural University, Harbin, China

**Keywords:** hydrogen peroxide, oxidative stress, piglet, *Ulva prolifera*, intestine

## Abstract

**Background:**

*Ulva prolifera* extract contains a variety of functional active substances. Whether these substances had any beneficial effects on the small intestine of weaned piglets under oxidative stress remained unknown.

**Method:**

We explored the effects of *U. prolifera* extract on oxidative stress and related mechanisms in weaned piglets and intestinal porcine epithelial cells (IPEC-J2) challenged with hydrogen peroxide.

**Results:**

*U. prolifera* extract was found to mainly consist of polyphenols and unsaturated fatty acids*. U. prolifera* extract increased total antioxidant capacity and superoxide dismutase (SOD) activity, while it decreased malondialdehyde content, in the serum of weaned piglets challenged with hydrogen peroxide. Moreover, *U. prolifera* extract increased mRNA expression of *SOD* and *catalase*, as well as the intestinal expression of nuclear NF-E2-related factor 2 (Nrf2), both *in vitro* and *in vivo*. Furthermore, *U. prolifera* extract decreased reactive oxygen species and improved mitochondrial respiration in IPEC-J2 cells treated with hydrogen peroxide. However, AMPK inhibition did not affect nuclear Nrf2 expression and only partially affected the effects of *U. prolifera* extract on oxidative stress.

**Conclusion:**

We suggest that *U. prolifera* extract alleviates oxidative stress *via* Nrf2 signaling, but independent of AMPK pathway in weaned piglets challenged with hydrogen peroxide. These results shed new insight into the potential applications of *U. prolifera* extract as a therapeutic agent for the prevention and treatment of oxidative stress-induced intestinal diseases.

## Introduction

*Ulva prolifera* is a common green algae that blooms in the Bohai and the Yellow Seas of China (1). In ancient China, *U. prolifera* is not only a food but also a pharmaceutical product for the treatment of diseases (2). In recent years, many components such as polyphenols, flavonoids, and polysaccharides have been extracted from *U. prolifera* (3–5). These components have been proven to widely exert antioxidant, anti-inflammatory, and hypolipidemic properties (1, 2, 6). Consequently, *U. prolifera* extract has the potential to be used for the prevention and treatment of diseases such as diabetes and non-alcoholic fatty liver diseases (4, 7). However, it remains unknown whether *U. prolifera* extract has any effects on oxidative damage in the intestine.

Redox homeostasis is a key factor in the maintenance of normal cellular and organic functions (8). The gastrointestinal tract is prone to oxidative damage as it faces a complicated luminal environment of ingested materials and microbial pathogens (9). The loss of redox homeostasis in the gastrointestinal tract due to oxidation-induced disruption can contribute to the development of various intestinal diseases such as ulcers, malignancies, and colitis (10). Hydrogen peroxide, a highly reactive oxygen species (ROS), causes the imbalance of intestinal cellular redox *in vivo* and *in vitro* (11). Previous studies have shown that hydrogen peroxide decreased the activity of antioxidant enzymes, increased ROS content and apoptosis level, and caused mitochondrial dysfunction in intestinal epithelial cells of weaned piglets (8, 11). These results suggested that weaned piglets challenged with hydrogen peroxide could provide appropriate dysfunctional intestine model for redox balance disruption. Additionally, many researchers suggested that the pig is a valuable resource for biomedical research, and experiments in pigs are much likely to be predictive of therapeutic treatments in humans (12). Because the pig is very similar to humans in terms of anatomy, genetics and physiology (13, 14).

Consequently, the present study was conducted to determine the effects of *U. prolifera* extract on oxidative stress and mitochondrial dysfunction, as well as its related mechanisms, in the intestine of hydrogen peroxide-challenged piglets. We expected that the results would shed new insight into the potential applications of *U. prolifera* extract as a therapeutic agent for the prevention and treatment of oxidative stress-induced intestinal diseases.

## Materials and Methods

### Sample Extraction

*U. prolifera* was collected in May 2018 from the coast of Beidaihe in the Bohai Sea. The collected *U. prolifera* was rinsed with running water and dried in the sun. One kilogram of dried *U. prolifera* was mixed with 5 L distilled water and extracted for 3 h at 90°C in an ultrasonic bath (200 W, 45 kHz). The supernatant was collected by ﬁltration through siliceous earth and then run through an adsorption chromatography column (120 cm L × 150 mm ID—Huamei Experiment Instrument Plant, Shanghai, China) packed with AB-8 macroporous adsorption resin. After initial elution with distilled water, the final extract (UE) was obtained by elution with 70% methanol, followed by freeze-drying. For sample preparation, 10 mg of UE powder was dissolved in 200 ml of distilled water. Then a 1 ml aliquot of the upper layer was filtered through a 0.22 µm nylon membrane prior to UHPLC/Q-TOF-MS analysis.

### UHPLC-Q-TOF-MS/MS Analysis

An Agilent 1290 Series UHPLC (Agilent, Palo Alto, CA, USA) was used and chromatography was performed in a ZORBAX Eclipse Plus C18 column (2.1 × 100 mm, 1.8 μm) (Agilent). The mobile phases consisted of water containing 0.1% formic acid (A) and acetonitrile (B). Gradient elution was performed with 5% B (0–1 min), 55% B (1–6 min), 95% B (6–20 min), 95% B (20–26 min), and 5% (1 min). Finally, the column was conditioned with 5% B for 8 min. The total run time was 35 min, with a flow rate of 0.25 ml/min, and an injection volume of 5 µl. The column temperature was 30°C.

MS was performed using an Agilent 6545 ESI-Q-TOF (Agilent). The optimum MS conditions consisted of a capillary voltage of 3500 V in negative ionization mode, a skimmer voltage of 65 V, and a fragmentor at 135 V. The gas temperature was 320°C, the drying gas flow rate was 8 L/min, and the nebulizer pressure was 35 psi. The sheath gas temperature was 350°C, and the sheath gas flow was 11 L/min. MS spectra were acquired at 100–1,700 m/z using an extended dynamic range at a scan rate of 2.0 spectra/s. A reference mass solution that contained reference ions (m/z 112.985587 and m/z 1033.988109) was used to maintain mass accuracy during the run. The MassHunter Workstation software (Agilent) was used to control the UHPLC/Q-TOF-MS system and to process recorded data, while the MassHunter Profiling software (Agilent) was used to screen the characteristic compound (15). The IM-MS Browser software (Agilent) and Traditional Chinese Medicine (TCM) database (Agilent) were used to identify the compound’s identity.

### Experimental Design

Thirty-two Landrace × Large White piglets (mean body weight of 6.81 ± 0.21 kg) were weaned at the age of 21 days, and randomly assigned to one of four treatment groups (n = 8/group): (1) Piglets fed a basal diet (CONT); (2) Piglets fed a basal diet supplemented with 0.1% extract of *U. prolifera* (UE group); (3) Piglets fed a basal diet and intraperitoneal injection of 10% hydrogen peroxide (1 ml/kg body weight) (HP); (4) Piglets fed a basal diet supplemented with 0.1% extract of *U. prolifera* and intraperitoneal injection of 10% hydrogen peroxide (UEH). The experiment lasted for 14 days and all piglets had free access to feed and drinking water. The diets for the UE and UEH groups were supplemented with *U. prolifera* extract daily. Hydrogen peroxide were administered intraperitoneally in the HP and UEH groups on days 8 and 11 as previously did (8). The composition and nutrient levels of the basal diet met the nutrient requirements listed in Nutritional Requirements of Swine (NRC, 2012) (**Supplementary Table 1**). Piglets were weighed on days 1 and 14, and their feed intake was recorded daily. Average daily feed intake, average daily weight gain, and the ratio of feed intake to weight gain, were calculated. The experimental protocol was approved by the Protocol Management and Review Committee of the Institute of Animal Husbandry, Heilongjiang Academy of Agricultural Sciences. Pigs were cared for and slaughtered according to the guidelines of Heilongjiang Academy of Agricultural Sciences (Harbin, China).

### Determination of Serum Antioxidative Enzymes and Malondialdehyde

Malondialdehyde (MDA) content, total antioxidant capacity (T-AOC), and superoxide dismutase (SOD) activities were analyzed in serum using commercial kits according to the manufacturer’s instructions (Beyotime Biotechnology, Shanghai, China).

### Cell Culture and Treatments

Intestinal porcine epithelial cells (IPEC-J2) were cultured in Dulbecco’s Modiﬁed Eagles Medium/Nutrient Mixture F-12 (Gibco, Carlsbad, CA, USA). After being seeded in 6-well plates and grown at ∼60−70% conﬂuence, cells cultured in fresh medium without fetal bovine serum (FBS) for 12 h were pretreated with *U. prolifera* extract (40 μg/ml) for 12 h, and then treated with 200 μM hydrogen peroxide for 6_ h_. For the inhibition of AMPK, 5 μM Compound C (Selleck Chemicals, Shanghai, China) was added to the medium for 6 h.

### RT-qPCR Analysis

Total RNA was extracted from ileum samples and then reverse-transcribed into cDNA using reverse transcriptase (Takara Bio, Tokyo, Japan). Pig-specific primers were designed using Primer 5.0 software (**Supplementary Table 2**). The mRNA expression level of the target gene was normalized using housekeeping genes *β-actin and GAPDH*. RT-PCR was performed as described previously (16, 17). Relative expression of the target gene in the treatment groups was expressed as a ratio to the expression of the target gene in the control group.

### Protein Qualification by the Wes Simple Western System

Protein qualification was performed using the Wes Simple Western System (ProteinSimple, San Jose, CA, USA), an automated process of capillary gel electrophoresis. Proteins were extracted from IPEC-J2 cells, mixed with Simple Western Sample Buffer, Master Mix, dithiothreitol, and fluorescent standards (ProteinSimple), and then loaded into Wes 25-well plates. Thereafter, primary and secondary antibodies, luminol-peroxide mixture, stacking gel matrix, and separation gel matrix were added to the appropriate wells. Primary antibodies used in the experiment included antibodies against Keap1, Nrf2, β-actin (Abcam, Cambridge, MA, USA); lamin B1, AMPK and phospho-AMPK (Bioss, Beijing, China). Results were obtained using the “gel view” function of the software (ProteinSimple). Total cellular protein expression was normalized to β-actin, while nuclear Nrf2 and Keap1 protein expression was normalized to lamin B1.

### Measurement of ROS in Mitochondria

IPEC-J2 cells seeded into confocal dishes were treated with 5 μM MitoSOX™ reagent working solution (Invitrogen, Shanghai, China) and incubated for 10 min at 37°C. Cells were then treated with anti-fluorescence quenching agent and observed using a ZEISS LSM 880 confocal microscope (ZEISS, Shanghai, China).

### Mitochondrial Membrane Potential Assay

Mitochondrial membrane potential was assayed by double fluorescence staining with JC-1 (Invitrogen, Shanghai, China). After being seeded into confocal dishes, the cells were incubated with 10 μg/ml JC-1 for 30 min, and then washed twice with PBS. Cells were then treated with anti-fluorescence quenching agent and observed using a ZEISS LSM 880 confocal microscope (ZEISS, Shanghai, China).

### Mitochondrial Respiration

Mitochondrial respiration was measured using an XF-24 Extracellular Flux Analyzer and a Cell Mito Stress Test Kit (Agilent, Beijing, China) according to the manufacturer’s instructions. The baseline oxygen-consumption rate (basal OCR), spare respiratory capacity, non-ATP-linked oxygen consumption (proton leakage), maximal respiratory capacity (maximal respiration), ATP-linked mitochondrial oxygen consumption (ATP production), and non-mitochondrial respiration were determined as previously described (18). Total cellular protein was analyzed for normalizing mitochondrial respiration rates.

### Immunofluorescent Assay

IPEC-J2 cells seeded into confocal dishes were fixed with 4% paraformaldehyde for 15 min, permeabilized with 0.3% Triton X-100 for 15 min. Cells were then blocked in 10% BSA for 20 min, and incubated with primary antibodies Keap1 and Nrf2 (Abcam, Cambridge, MA, USA) overnight at 4°C. After being washed three times with PBS, cells were incubated with secondary antibodies for 1 h. The nuclear DNA was labeled with 4’,6-Diamidino-2-phenylindole (Sigma-Aldrich, St. Louis, MO, USA) for 2 min, and cells were then treated with anti-fluorescence quenching agent and observed using a ZEISS LSM 880 confocal microscope (ZEISS, Shanghai, China).

### AMPK Activity Determination

AMPK activity was determined using AMPK Kinase Assay Kit (CycLex, Tokyo, Japan) as previously described (19). Briefly, IPEC-J2 cells were collected and lysed. Then, supernatant was obtained by centrifugation at 12,000 *g* and 4°C for 15 min, for the determination of the relative AMPK activity.

### Statistical Analysis

Statistical analysis was performed by one-way ANOVA followed by Student-Newman-Keuls *post hoc* test using data statistics software SPSS 18.0. All the measurement data was expressed as the means ± standard error (SEM). P < 0.05 was considered statistically significant.

## Results

### Characterization of Components of *U. Prolifera* Extract

By screening with Agilent’s TCM database, *U. prolifera* extract was found to mainly consist of polyphenols and unsaturated fatty acids (**Table 1**).

**Table 1:** Identiﬁcation of compounds extracted from *Ulva prolifera* using hot water extraction method.

Compound	Formula	(M-H)-	Rt (min)
Kaempferol	C_15_H_10_O_6_	286.1474	12.70
Neoeriocitrin	C_27_H_32_O15	597.1245	6.59
20(R)-Ginsenoside-Rh2	C_36_H_62_O_8_	622.4424	9.313
Uvaribonone	C_39_H_68_O_8_	664.4891	7.24
Chlorogenic acid	C_16_H_18_O_9_	354.31	20.12
Methyl-n-nonylketone	C_11_H_22_O	170.1675	13.9
Oleanolic acid-28-O-beta-D-glucopyranoside	C_36_H_58_O_8_	618.4109	8.996
alpha-Eleostearic acid	C_18_H_30_O_2_	278.225	22.147
cis-9,cis-12-Linoleic acid	C_18_H_32_O_2_	280.2405	24.075
Clupanodonic acid	C_18_H_28_O_2_	276.2089	20.561
Palmitoleic acid	C_16_H_30_O_2_	254.2246	23.733
Unnamed	C_20_H_22_N_6_O	362.1862	9.692
Unnamed	C_18_H_26_N_10_O	398.2286	9.326
Unnamed	C_37_H_63_N_11_O_5_	741.5012	7.24

Ulva prolifera extracts were obtained using hot water extraction method, and the extracts were analyzed by UHPLC-Q-TOF-MS/MS and identified according to the IM-MS Browser software (Agilent) and Traditional Chinese Medicine (TCM) database.

### *U. prolifera* Extract Improved Growth Performance and Alleviated Oxidative Stress in Weaned Piglets Challenged With Hydrogen Peroxide

**Figure 1:**
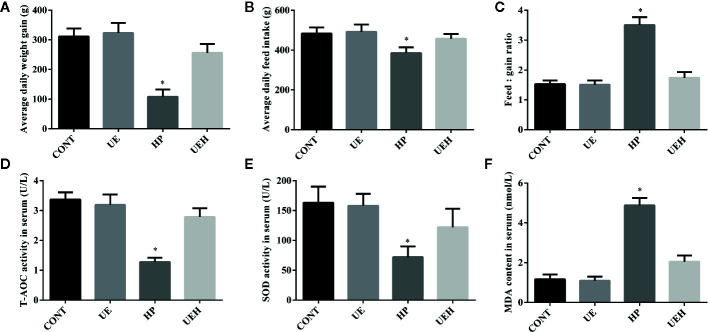
*Ulva prolifera* extract improved growth performance and alleviated oxidative stress in weaned piglets challenged with hydrogen peroxide. **(A)** Average daily weight gain; **(B)** Average daily feed intake; **(C)** Feed: gain ratio; **(D)** T-AOC activity in serum; **(E)** SOD activity in serum; **(F)** MDA content in serum. T-AOC, total antioxidant ability; SOD, superoxide dismutase; MDA, Malondialdehyde. CONT, piglets fed a basal diet and administrated intraperitoneally with saline (1 ml/kg BW); UE, piglets fed a basal diet and supplemented with 0.1% *U. prolifera* extract; HP, piglets fed a basal diet and administrated intraperitoneally with 10% hydrogen peroxide (1 ml/kg body weight); UEH, piglets fed a basal diet supplemented with 0.1% *U. prolifera* extract, and administrated intraperitoneally with 10% hydrogen peroxide. Values are expressed as mean ± SEM, n = 8; *p < 0.05.

Piglets challenged with hydrogen peroxide showed decreased average daily weight gain and feed intake, while showing an increased ratio of feed intake to weight gain, indicating reduced growth performance (**Figures 1A–C**). When the piglets’ diet was supplemented with *U. prolifera* extract, the growth performance was unchanged compared with control piglets, even if the piglets were challenged with hydrogen peroxide. Compared with control piglets, piglets challenged with hydrogen peroxide had lower T-AOC and SOD activities, but higher MDA content in serum, while no such changes were observed in piglets supplemented with *U. prolifera* extract (**Figures 1D–F**).

### *U. prolifera* Extract Alleviated Oxidative Stress and Activated AMPK/Nrf2 Signaling in the Ileum of Weaned Piglets Challenged With Hydrogen Peroxide

**Figure 2:**
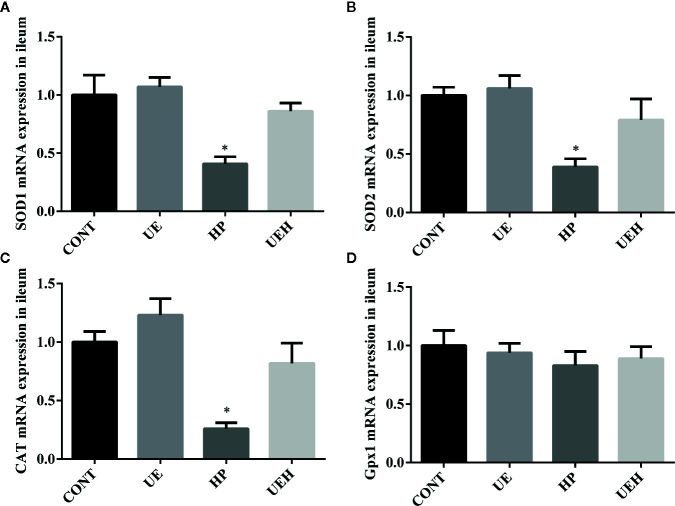
*Ulva prolifera* extract promoted expression of the antioxidant genes in the ileum of weaned piglets challenged with hydrogen peroxide. Gene expression of *SOD1* **(A)**, *SOD2* **(B)**, *CAT* **(C)**, and *Gpx1* **(D)** in ileum. *SOD*, superoxide dismutase; *CAT*, catalase; *Gpx1*, glutathione peroxidase 1. CONT, piglets fed a basal diet and administrated intraperitoneally with saline (1 ml/kg BW); UE, piglets fed a basal diet and supplemented with 0.1% *U. prolifera* extract; HP, piglets fed a basal diet and administrated intraperitoneally with 10% hydrogen peroxide (1 ml/kg body weight); UEH, piglets fed a basal diet supplemented with 0.1% *U. prolifera* extract, and administrated intraperitoneally with 10% hydrogen peroxide. Values are expressed as mean ± SEM, n = 8; *p < 0.05.

**Figure 3:**
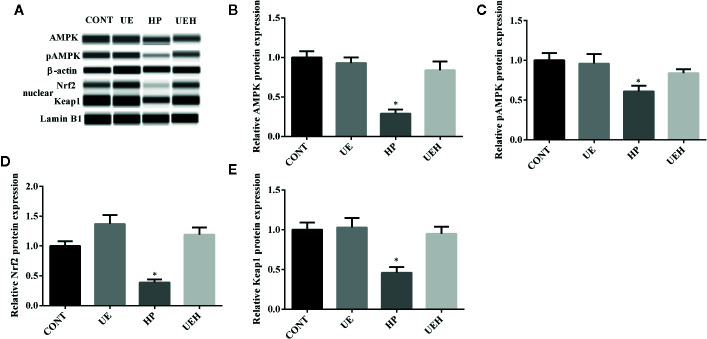
*Ulva prolifera* extract activated the AMPK/Nrf2 signaling pathway in the ileum of weaned piglets challenged with hydrogen peroxide. **(A)** Western blotting results; **(B)** AMPK abundance relative to β-actin; **(C)** pAMPK abundance relative to AMPK; **(D)** Nuclear Nrf2 abundance relative to Lamin B1; **(E)** Nuclear Keap1 abundance relative to Lamin B1. CONT, piglets fed a basal diet and administrated intraperitoneally with saline (1 ml/kg BW); UE, piglets fed a basal diet and supplemented with 0.1% *U. prolifera* extract; HP, piglets fed a basal diet and administrated intraperitoneally with 10% hydrogen peroxide (1 ml/kg body weight); UEH, piglets fed a basal diet supplemented with 0.1% *U. prolifera* extract, and administrated intraperitoneally with 10% hydrogen peroxide. AMPK, AMP-activated protein kinase; Nrf2, NF-E2-related factor 2; Keap1, inhibitor of Nrf2. Values are expressed as mean ± SEM, n = 3; *p < 0.05.

Compared with those in control piglets, piglets challenged with hydrogen peroxide had higher *SOD1*, *SOD2*, and *CAT* mRNA expression in the ileum, while no changes were observed in piglets supplemented with *U. prolifera* extract (**Figures 2A–C**). No change in *Gpx1* mRNA expression in the piglet ileum was observed across the four treatments (**Figure 2D**). Hydrogen peroxide challenge caused significant decreases in protein expression of AMPK and phosphorylated AMPK, as well as nuclear Nrf2 and Keap1, in the weaned piglet ileum, while *U. prolifera* extract significantly prevented these changes (**Figure 3**).

### *U. prolifera* Extract Alleviated Oxidative Stress in the IPEC-J2 Cells Treated With Hydrogen Peroxide

**Figure 4:**
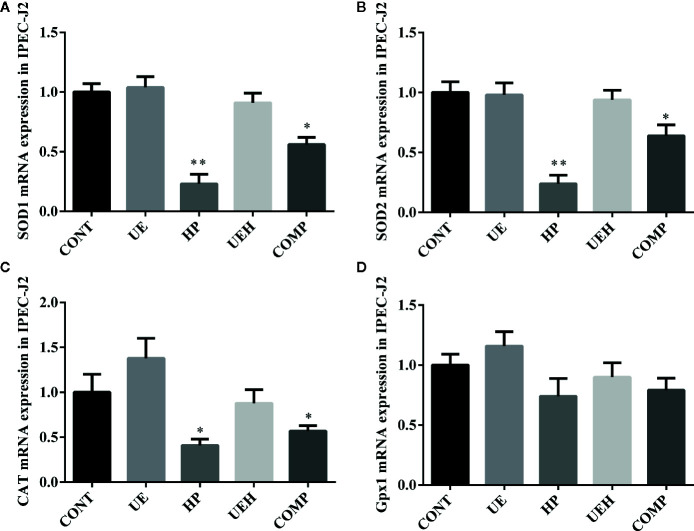
*Ulva prolifera* extract promoted expression of the antioxidant genes in IPEC-J2 cells treated with hydrogen peroxide. Gene expression of *SOD1* **(A)**, *SOD2* **(B)**, *CAT* **(C)**, and *Gpx1* **(D)** in IPEC-J2 cells. *SOD*, superoxide dismutase; *CAT*, catalase; *Gpx1*, glutathione peroxidase 1. CONT, control cells; UE, cells treated with 40 μg/ml *U. prolifera* extract; HP, cells treated with 200 μM hydrogen peroxide; UEH, cells treated with 40 μg/ml *U. prolifera* extract and 200 μM hydrogen peroxide; COMP, cells treated with 40 μg/ml *U. prolifera* extract, 200 μM hydrogen peroxide, and 5 μM compound C. Values are expressed as mean ± SEM, n = 6; *p < 0.05, **p < 0.01.

**Figure 5:**
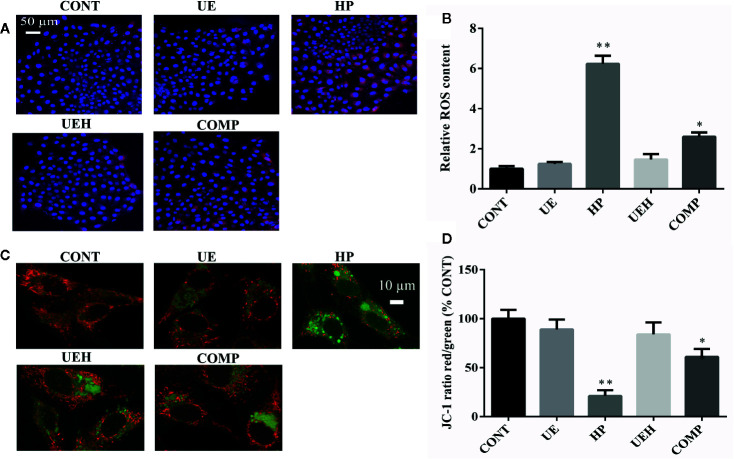
*Ulva prolifera* extract decreased ROS content and alleviated oxidative stress in IPEC-J2 cells treated with hydrogen peroxide. **(A)** ROS staining (red, ROS; blue, DAPI); **(B)** Relative ROS content; **(C)** JC-1 staining (red, aggregate; green, monomer); **(D)** JC-1 ratio (red to green). ROS, reactive oxygen species. CONT, control cells; UE, cells treated with 40 μg/ml *U. prolifera* extract; HP, cells treated with 200 μM hydrogen peroxide; UEH, cells treated with 40 μg/ml *U. prolifera* extract and 200 μM hydrogen peroxide; COMP, cells treated with 40 μg/ml *U. prolifera* extract, 200 μM hydrogen peroxide and 5 μM compound C. Values are expressed as mean ± SEM, n = 3; *p < 0.05, **p < 0.01.

Hydrogen peroxide treatment caused significant decreases in mRNA expression of *SOD1*, *SOD2*, and *CAT* in IPEC-J2 cells, while *U. prolifera* extract significantly prevented these changes (**Figures 4A–C**). Moreover, cells further treated with compound C, an inhibitor of AMPK, showed significantly less effect of *U. prolifera* extract, even though mRNA expression of *SOD1* and *SOD2* remained significantly higher than observed in hydrogen peroxide-treated cells. No changes in *Gpx1* mRNA expression were observed in IPEC-J2 cells across the five treatments (**Figure 4D**). ROS content was significantly higher in cells treated with hydrogen peroxide, while no difference in ROS content was observed when the cells were treated with *U. prolifera* extract (**Figures 5A, B**). ROS content in hydrogen peroxide-induced cells treated with both *U. prolifera* extract and compound C was significantly higher compared with control cells, but ROS content was lower when compared with cells treated only with hydrogen peroxide.

JC-1 staining, as a marker of mitochondrial membrane potential, was further used to assess mitochondrial stress. The formation of JC-1 aggregates (red fluorescence) reflects lower stress while JC-1 monomers (green fluorescence) suggest higher stress. Hydrogen peroxide induced a decrease in the ratio of red to green fluorescence, while *U. prolifera* extract diminished this ratio decrease (**Figures 5C, D**). The ratio of red to green fluorescence in cells treated with compound C was higher than the ratio observed in control cells and *U. prolifera* extract-treated cells, but lower than observed in hydrogen peroxide-treated cells.

### *U. prolifera* Extract Promoted Mitochondrial Respiration in IPEC-J2 Cells Treated With Hydrogen Peroxide

**Figure 6:**
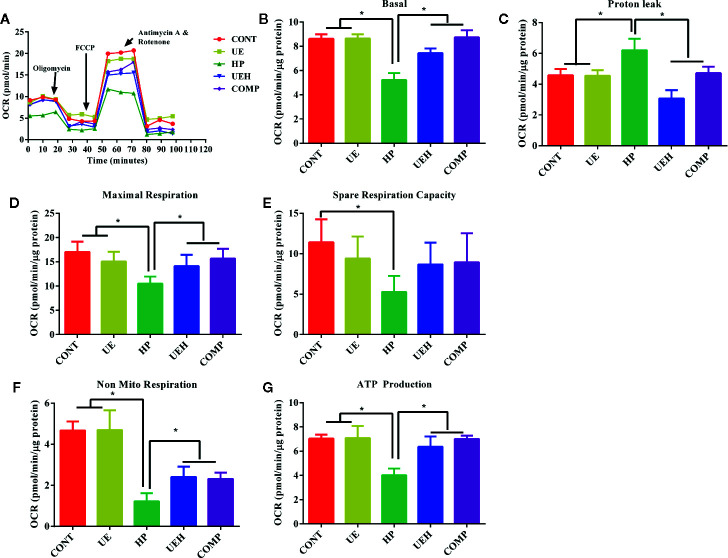
*Ulva prolifera* extract promoted mitochondrial respiration in IPEC-J2 cells treated with hydrogen peroxide. **(A)** mitochondrial respiration curve; **(B)** Basal respiration; **(C)** Proton leak; **(D)** Maximal respiration; **(E)** Spare respiration capacity; **(F)** Non mitochondrial respiration; **(G)** ATP production. CONT, control cells; UE, cells treated with 40 μg/ml *U. prolifera* extract; HP, cells treated with 200 μM hydrogen peroxide; UEH, cells treated with 40 μg/ml *U. prolifera* extract and 200 μM hydrogen peroxide; COMP, cells treated with 40 μg/ml *U. prolifera* extract, 200 μM hydrogen peroxide, and 5 μM compound C. Values are expressed as mean ± SEM, n = 3; *p < 0.05.

The results of mitochondrial respiration determination are shown in **Figure 6A**. Challenge with hydrogen peroxide significantly decreased basal OCR, maximal respiration, spare respiration capacity, non-mitochondrial respiration, and ATP production, and increased proton leak, while *U. prolifera* extract alleviated these changes in IPEC-J2 cells (**Figures 6B–G**). Cells further treated with compound C did not show any change in these parameters, except that spare respiration capacity was not altered between cells treated with compound C and cells treated with hydrogen peroxide only.

### *U. prolifera* Extract Activated AMPK/Nrf2 Signaling in the IPEC-J2 Cells Treated With Hydrogen Peroxide

**Figure 7:**
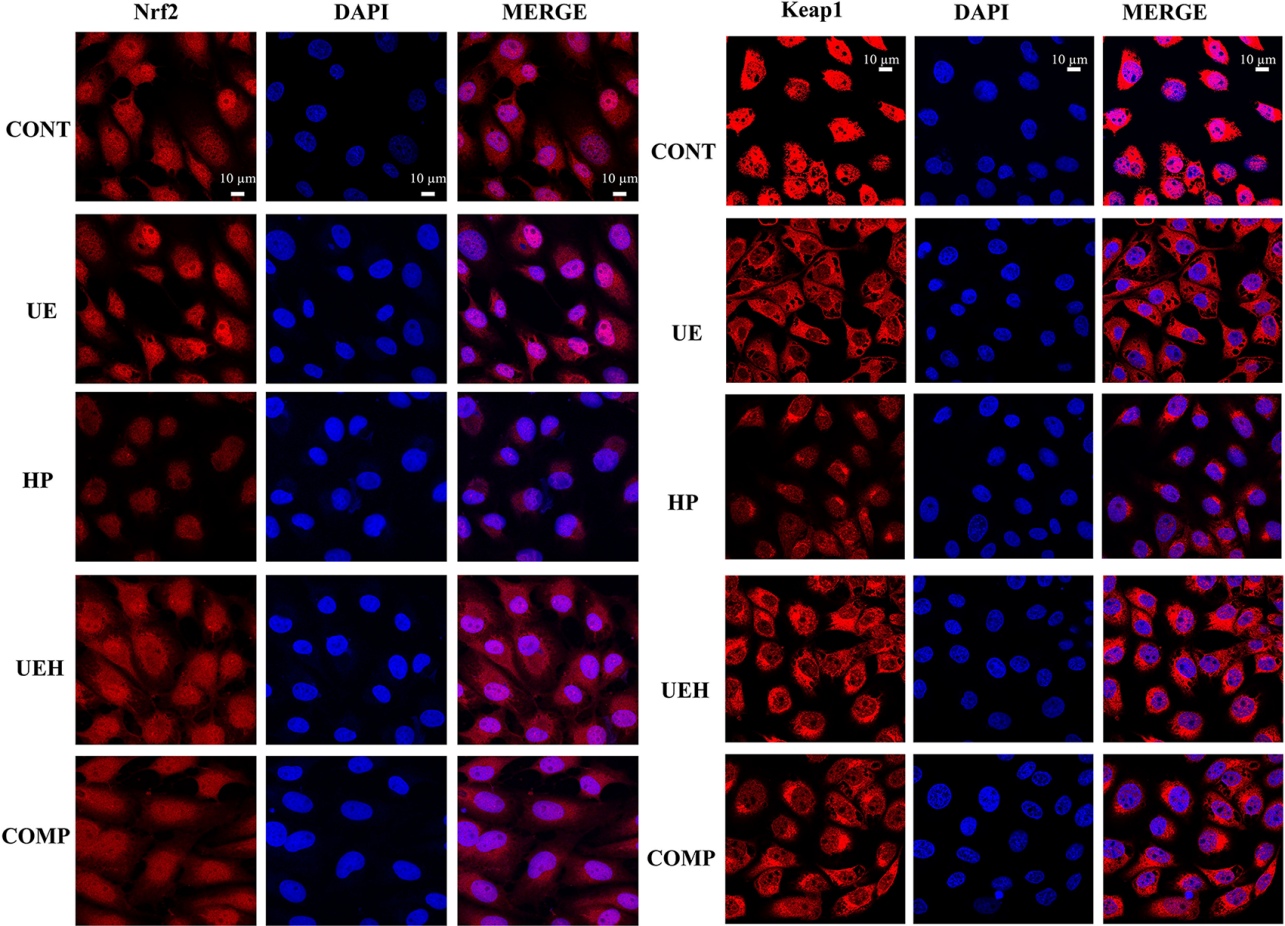
*Ulva prolifera* extract increased expression of Nrf2 and Keap1 in IPEC-J2 cells treated with hydrogen peroxide. CONT, control cells; UE, cells treated with 40 μg/ml *U. prolifera* extract; HP, cells treated with 200 μM hydrogen peroxide; UEH, cells treated with 40 μg/ml *U. prolifera* extract and 200 μM hydrogen peroxide; COMP, cells treated with 40 μg/ml *U. prolifera* extract, 200 μM hydrogen peroxide, and 5 μM compound C. Nrf2, NF-E2-related factor 2; Keap1, inhibitor of Nrf2.

**Figure 8:**
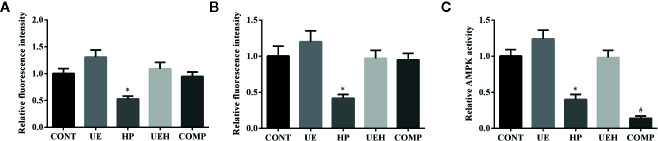
*Ulva prolifera* extract activated AMPK/Nrf2 signaling pathway in IPEC-J2 cells treated with hydrogen peroxide. Relative fluorescence intensity of Nrf2 **(A)** and Keap1 **(B)** based on the results of **Figure 7**; **(C)** Relative AMPK activity. CONT, control cells; UE, cells treated with 40 μg/ml *U. prolifera* extract; HP, cells treated with 200 μM hydrogen peroxide; UEH, cells treated with 40 μg/ml *U. prolifera* extract and 200 μM hydrogen peroxide; COMP, cells treated with 40 μg/ml *U._ prolifera* extract, 200 μM hydrogen peroxide, and 5 μM compound C. AMPK, AMP-activated protein kinase; Nrf2, NF-E2-related factor 2; Keap1, inhibitor of Nrf2. Values are expressed as mean ± SEM, n = 3; *p < 0.05 relative to CONT, UE, and UEH; ^#^p < 0.05 relative to HP.

The results of immunofluorescent assay show that nuclear Nrf2 and Keap1 expression was decreased by hydrogen peroxide, while *U. prolifera* extract alleviated these changes in IPEC-J2 cells (**Figures 7** and **8**). Compound C treatment did not alter the effects of *U. prolifera* extract on Nrf2 and Keap1 expression. We determined the activity of AMPK to further investigate any changes to AMPK signaling. The results showed that hydrogen peroxide caused significant decrease of AMPK activity, and this decrease was alleviated by *U. prolifera* extract (**Figure 8**).

## Discussion

*U. prolifera*, the major causative species of green tides, has bloomed in the Yellow Sea since 2008 and bloomed in the Bohai Sea in 2015, leading to marine ecological disasters as well as great losses to tourism and aquaculture (1). However, many bioactive materials in *U. prolifera* are considered nutritious and have functional activities. In the present study, we extracted bioactive constituents from *U. prolifera* using hot water extraction, analyzed the components by UHPLC-Q-TOF-MS/MS analysis, and screened the components using Agilent’s TCM database. Except that other researchers who extracted polysaccharides and flavonoids in the green algae, *U. prolifera* extract was found to contain polyphenols and unsaturated fatty acids in our study (2, 4). By supplementation with *U. prolifera* extract in hydrogen peroxide-challenged piglets, interestingly, we found that *U. prolifera* extract protected the intestine from oxidative damage. *U. prolifera* extract also improved mitochondrial function and activated the AMPK/Nrf2 signaling pathway in intestinal porcine epithelial cells.

Hydrogen peroxide can diffuse across the cell membrane and penetrate throughout the mitochondria (20). Overaccumulation of hydrogen peroxide and its metabolites, such as hydroxyl radicals, oxidizes carbohydrates, lipids, nucleic acids, and proteins, resulting in cellular injury that includes mitochondrial dysfunction. This state of oxidative stress further impairs organ function. Previous studies have shown that hydrogen peroxide not only inhibited SOD and T-AOC activities but also increased MDA content, indicating an oxidative imbalance in weaned piglets challenged with hydrogen peroxide (8, 20–22). Additionally, hydrogen peroxide treatment caused morphological injury and mitochondrial dysfunction, and promoted apoptosis in intestinal porcine epithelial cells (11). These results suggested that weaned piglets challenged with hydrogen peroxide would be a suitable model for studying intestinal oxidative damage and ROS-induced intestinal diseases.

In the present study, we used the described piglet model and our results suggested that the weaned piglets were under oxidative stress after peritoneal injections with hydrogen peroxide, which is in agreement with previous studies (8, 11). Interestingly, *U. prolifera* extract showed a strong protective effect against hydrogen peroxide-induced oxidative responses in weaned piglets. Importantly, *U. prolifera* extract exerted beneficial effects on mitochondrial function *via* preventing the impairment of ATP-linked respiration and maximal respiratory capacity from hydrogen peroxide challenge, according to the results of the mitochondrial respiration assay (23). Mitochondria play critical roles in generating, sensing, and scavenging ROS (24). We proposed that *U. prolifera* extract may help maintain mitochondrial function by improving mitochondrial metabolism of ROS when the intestinal porcine epithelial cells were challenged with hydrogen peroxide. This was evidenced by lower mitochondrial ROS content and higher JC-1 red to green fluorescence ratios in cells treated with hydrogen peroxide and *U. prolifera* extract, which suggested lower mitochondrial stress.

AMP-activated protein kinase (AMPK), a cellular sensor of redox balance, is involved in the regulation of cellular antioxidant response. It has been suggested that AMPK is an upstream regulator of Nrf2 and its activation causes nuclear accumulation of Nrf2 (25). However, our results suggested that inhibition of AMPK did not affect nuclear Nrf2 expression, although the effects of *U. prolifera* extract on oxidative stress was partially affected. Based on these results, we suggest that *U. prolifera* extract may exert direct influence on the Nrf2 pathway or has activated other upstream regulators of Nrf2. The Nrf2/Keap1 pathway is considered the cell’s most important antioxidant system (26). The initial effect of ROS on the Nrf2/Keap1 pathway is to promote the dissociation of Nrf2 from the Nrf2/Keap1 complex, which then translocates into the nucleus to promote the expression of its downstream antioxidant enzyme genes. However, continuously increased ROS further exerts accumulative effects that lead to the degradation of nuclear Nrf2, switching off the target gene expression activation. Hydrogen peroxide was hypothesized to influence the formation of the Nrf2/Keap1 complex and translocation of Nrf2 by promoting the production of ROS (18, 27). We observed the accumulative negative effects of hydrogen peroxide in the intestine of weaned piglets. Importantly, *U. prolifera* extract protected the intestinal porcine epithelial cells from a dysfunctional Nrf2/Keap1 signaling pathway. We suggest that *U. prolifera* extract exerted these protective effects *via* the elimination of ROS overproduction.

In conclusion, our results suggested that *U. prolifera* extract alleviated oxidative stress and improved mitochondrial function in the small intestine of weaned piglets challenged with hydrogen peroxide. Moreover, the Nrf2 signaling pathway played a critical role in mediating these effects. Our results provide potential applications of *U. prolifera* in animal feed and for medical purposes. These alternative uses indicate effective methods to bring the environmental damage under control and to profit from green tide events.

## Data Availability Statement

The raw data supporting the conclusion of this article will be made available by the authors, without undue reservation.

## Ethics Statement

The animal study was reviewed and approved by Ethics of Animal Experiments of Heilongjiang Academy of Agricultural Sciences.

## Author Contributions

YF, FL, and DL designed the experiment and drafted the manuscript. HC, XH, WW, XL, and HZ carried out the animal trials and sample analysis. XL did data analysis work. FL and DL were responsible for the integrity of the work as a whole. All authors contributed to the article and approved the submitted version.

## Funding

This work was supported by the Cooperative Innovation and Popularization System of Modern Agricultural Technology of Heilongjiang Provincial Pig Production and the Earmarked Fund for China Agriculture Research System (CARS-36).

## Conflict of Interest

The authors declare that the research was conducted in the absence of any commercial or financial relationships that could be construed as a potential conflict of interest.

## References

[1] Song W Wang Z Zhang X Li Y Ethanol extract from Ulva prolifera prevents high-fat diet-induced insulin resistance, oxidative stress, and inflammation response in mice. BioMed Res Int (2018) 2018:1374565. 10.1155/2018/1374565 29511669 PMC5817197

[2] Teng Z Qian L Zhou Y Hypolipidemic activity of the polysaccharides from Enteromorpha prolifera. Int J Biol Macromol (2013) 62:254–6. 10.1016/j.ijbiomac.2013.09.010 24060283

[3] Shao LL Xu J Shi MJ Wang XL Li YT Kong LM Preparation, antioxidant and antimicrobial evaluation of hydroxamated degraded polysaccharides from Enteromorpha prolifera. Food Chem (2017) 237:481–7. 10.1016/j.foodchem.2017.05.119 28764023

[4] Yan X Yang C Lin G Chen Y Miao S Liu B Antidiabetic potential of green seaweed enteromorpha prolifera flavonoids regulating insulin signaling pathway and gut microbiota in Type 2 diabetic mice. J Food Sci (2019) 84(1):165–73. 10.1111/1750-3841.14415 30569533

[5] Lin G Liu X Yan X Liu D Yang C Liu B Role of green macroalgae enteromorpha prolifera polyphenols in the modulation of gene expression and intestinal microflora profiles in Type 2 diabetic mice. Int J Mol Sci (2018) 20(1):25. 10.3390/ijms20010025 PMC6337142 30577594

[6] Cho M Lee HS Kang IJ Won MH You S Antioxidant properties of extract and fractions from Enteromorpha prolifera, a type of green seaweed. Food Chem (2011) 127(3):999–1006. 10.1016/j.foodchem.2011.01.072 25214089

[7] Ren R Gong J Zhao Y Zhuang X Ye Y Lin W Sulfated polysaccharides from Enteromorpha prolifera suppress SREBP-2 and HMG-CoA reductase expression and attenuate non-alcoholic fatty liver disease induced by a high-fat diet. Food Funct (2017) 8(5):1899–904. 10.1039/c7fo00103g 28429814

[8] Duan J Yin J Ren W Liu T Cui Z Huang X Dietary supplementation with L-glutamate and L-aspartate alleviates oxidative stress in weaned piglets challenged with hydrogen peroxide. Amino Acids (2016) 48(1):53–64. 10.1007/s00726-015-2065-3 26255283

[9] Circu ML Aw TY Intestinal redox biology and oxidative stress. Semin Cell Dev Biol (2012) 23(7):729–37. 10.1016/j.semcdb.2012.03.014 PMC3396776 22484611

[10] Bhattacharyya A Chattopadhyay R Mitra S Crowe SE Oxidative stress: an essential factor in the pathogenesis of gastrointestinal mucosal diseases. Physiol Rev (2014) 94(2):329–54. 10.1152/physrev.00040.2012 PMC4044300 24692350

[11] Yin J Wu M Li Y Ren W Xiao H Chen S Toxicity assessment of hydrogen peroxide on Toll-like receptor system, apoptosis, and mitochondrial respiration in piglets and IPEC-J2 cells. Oncotarget (2017) 8(2):3124–31. 10.18632/oncotarget.13844 PMC5356869 27966452

[12] Nossol C Barta-Boszormenyi A Kahlert S Zuschratter W Faber-Zuschratter H Reinhardt N Comparing two intestinal porcine epithelial cell lines (IPECs): morphological differentiation, function and metabolism. PLoS One (2015) 10(7):e0132323. 10.1371/journal.pone.0132323 26147118 PMC4493080

[13] Flisikowska T Kind A Schnieke A Genetically modified pigs to model human diseases. J Appl Genet (2014) 55(1):53–64. 10.1007/s13353-013-0182-9 24234401

[14] Meurens F Summerfield A Nauwynck H Saif L Gerdts V The pig: a model for human infectious diseases. Trends Microbiol (2012) 20(1):50–7. 10.1016/j.tim.2011.11.002 PMC7173122 22153753

[15] Jin XL Wang K Li QQ Tian WL Xue XF Wu LM Antioxidant and anti-inflammatory effects of Chinese propolis during palmitic acid-induced lipotoxicity in cultured hepatocytes. J Funct Foods (2017) 34:216–23. 10.1016/j.jff.2017.04.039

[16] Zhou X He L Zuo S Zhang Y Wan D Long C Serine prevented high-fat diet-induced oxidative stress by activating AMPK and epigenetically modulating the expression of glutathione synthesis-related genes. Biochim Biophys Acta Mol Basis Dis (2018) 1864(2):488–98. 10.1016/j.bbadis.2017.11.009 29158183

[17] Zhou X He L Wu C Zhang Y Wu X Yin Y Serine alleviates oxidative stress via supporting glutathione synthesis and methionine cycle in mice. Mol Nutr Food Res (2017) 61(11):1700262. 10.1002/mnfr.201700262 28759161

[18] He L Wu J Tang W Zhou X Lin Q Luo F Prevention of Oxidative Stress by alpha-Ketoglutarate via Activation of CAR Signaling and Modulation of the Expression of Key Antioxidant-Associated Targets in Vivo and in Vitro. J Agric Food Chem (2018) 66(43):11273–83. 10.1021/acs.jafc.8b04470 30346763

[19] He L Long J Zhou X Liu Y Li T Wu X Serine is required for the maintenance of redox balance and proliferation in the intestine under oxidative stress. FASEB J (2020) 34(3):4702–17. 10.1096/fj.201902690R 32030825

[20] Yin J Duan JL Cui ZJ Ren WK Li TJ Yin YL Hydrogen peroxide-induced oxidative stress activates NF-kappa B and Nrf2/Keap1 signals and triggers autophagy in piglets. Rsc Adv (2015) 5(20):15479–86. 10.1039/c4ra13557a

[21] Duan JL Yin J Ren WK Wu MM Chen S Cui ZJ Pyrrolidine dithiocarbamate restores gastric damages and suppressive autophagy induced by hydrogen peroxide. Free Radic Res (2015) 49(2):210–8. 10.3109/10715762.2014.993627 25471085

[22] Wang K Jin X Li Q Sawaya A Le Leu RK Conlon MA Propolis from different geographic origins decreases intestinal inflammation and Bacteroides spp. populations in a model of DSS-induced colitis. Mol Nutr Food Res (2018) 62(17):e1800080. 10.1002/mnfr.201800080 29889351

[23] Ferramosca A Pinto Provenzano S Montagna DD Coppola L Zara V Oxidative stress negatively affects human sperm mitochondrial respiration. Urology (2013) 82(1):78–83. 10.1016/j.urology.2013.03.058 23806394

[24] Starkov AA Andreyev AY Zhang SF Starkova NN Korneeva M Syromyatnikov M Scavenging of H_2_O_2_ by mouse brain mitochondria. J Bioenerg Biomembr (2014) 46(6):471–7. 10.1007/s10863-014-9581-9 PMC4634880 25248416

[25] Joo MS Kim WD Lee KY Kim JH Koo JH Kim SG AMPK facilitates nuclear accumulation of Nrf2 by phosphorylating at serine 550. Mol Cell Biol (2016) 36(14):1931–42. 10.1128/Mcb.00118-16 PMC4936058 27161318

[26] Kaspar JW Niture SK Jaiswal AK Nrf2:INrf2 (Keap1) signaling in oxidative stress. Free Radic Biol Med (2009) 47(9):1304–9. 10.1016/j.freeradbiomed.2009.07.035 PMC2763938 19666107

[27] Jaiswal AK Nrf2 signaling in coordinated activation of antioxidant gene expression. Free Radic Biol Med (2004) 36(10):1199–207. 10.1016/j.freeradbiomed.2004.02.074 15110384

