# Supplementary material for: Ulva prolifera Extract Alleviates Intestinal Oxidative Stress via Nrf2 Signaling in Weaned Piglets Challenged With Hydrogen Peroxide
Source: Front Immunol. 2020 Oct 30;11:599735. doi: 10.3389/fimmu.2020.599735 (PMC7661684; doi:10.3389/fimmu.2020.599735)
Supplement: Supplementary file 1 [file Table_1.docx]

**Supplementary Table 1 Ingredient and nutrient levels of diets for piglets.**

| Ingredient^#^ | Basal diet (%) |
| --- | --- |
| Soybean | 22 |
| Corn | 56.77 |
| Rice bran meal | 5 |
| Extruded corn | 5 |
| Broken rice | 6 |
| Fish meal | 2 |
| Calcium hydrophosphate | 1 |
| L-lysine | 0.4 |
| L- threonine | 0.1 |
| DL-methionine | 0.23 |
| Calcium lactate | 0.3 |
| Zinc oxide | 0.1 |
| Limestone | 0.22 |
| Premix including minerals and vitamins^*^ | 0.78 |
| Total | 100 |
| Analyzed value |  |
| Dry matter | 86.92 |
| Moisture | 13.08 |
| Crude protein | 18.41 |
| Ether extract | 3.19 |
| L-lysine | 1.004 |
| L- threonine | 0.75 |
| DL-methionine | 0.49 |
| L-cysteine | 0.26 |

^#^The ingredient and nutrient levels of the basal diet met the nutrient requirements listed in Nutritional Requirements of Swine (NRC, 2012).

^*^Content of premix (/kg per diet): Cu 10 mg (copper sulfate); Mn 10 mg (manganese sulfate); Fe 100 mg (iron sulfate); Se 0.3 mg (sodium selenite); I 0.3 mg (calcium iodate);Vitamin A 7500 IU; vitamin D3 750 IU; vitamin E 25 IU; vitamin K3 2.0 mg; vitamin B1 1.875 mg; vitamin B2 3.75 mg; vitamin B6 2.19 mg; vitamin B12 0.025 mg; nicotinic acid 25 mg; D-pantothenic acid 15.6 mg; folic acid 2.0 mg; biotin 0.1875 mg.

**Supplementary Table 2 Primer sequences for RT-qPCR**

| Gene | 5’-3’ Primer sequence |
| --- | --- |
| *SOD1* | F: GCGAGTCATGGCGACGAA |
|  | R: CACAGTGGCCACACCATCTT |
| *SOD2* | F: GGCCTACGTGAACAACCTGA |
|  | R: TGATTGATGTGGCCTCCACC |
| *CAT* | F: CCTGCAACGTTCTGTAAGGC |
|  | R: GCTTCATCTGGTCACTGGCT |
| *Gpx1* | F: CTAGCAGTGCCTAGAGTGCC |
|  | R: CGCCCATCTCAGGGGATTTT |
| *β-actin* | F: CTCCAGAGCGCAAGTACTCC |
|  | R: GCGTCCATCACAGCTTCTCA |
| *GAPDH* | F: TCGGAGTGAACGGATTTGGC |
|  | R: TGACAAGCTTCCCGTTCTCC |

*Gpx1*, glutathione peroxidase 1; *SOD*, superoxide dismutase; *CAT*, catalase.

**Supplementary Figure 1 Full images for results of Figure 3A.**


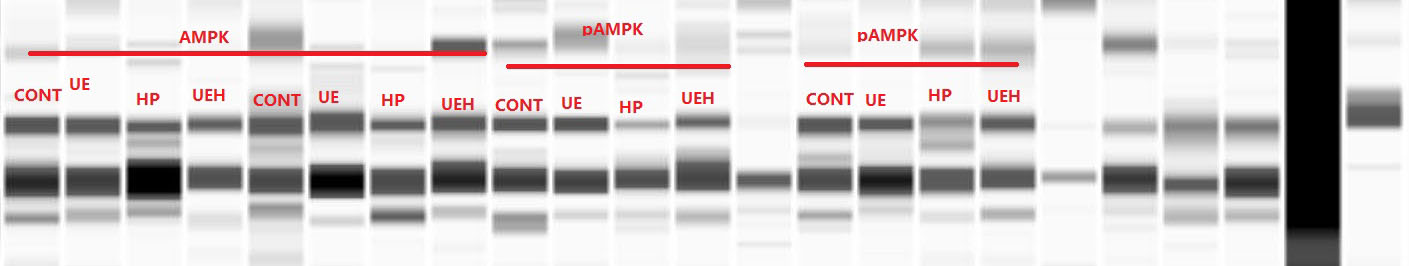


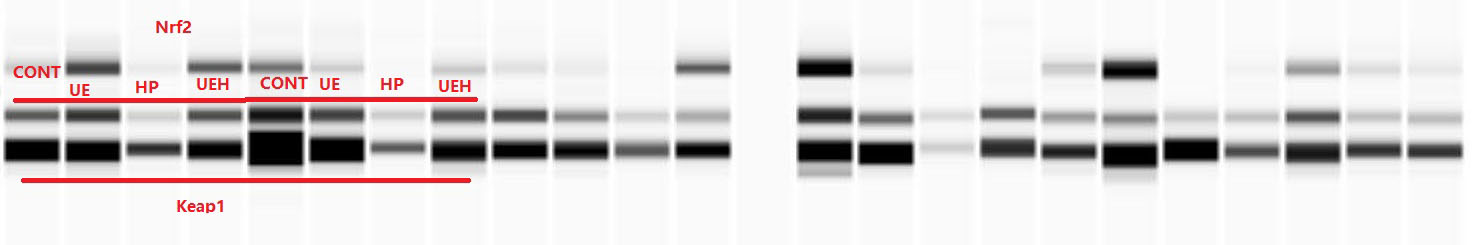


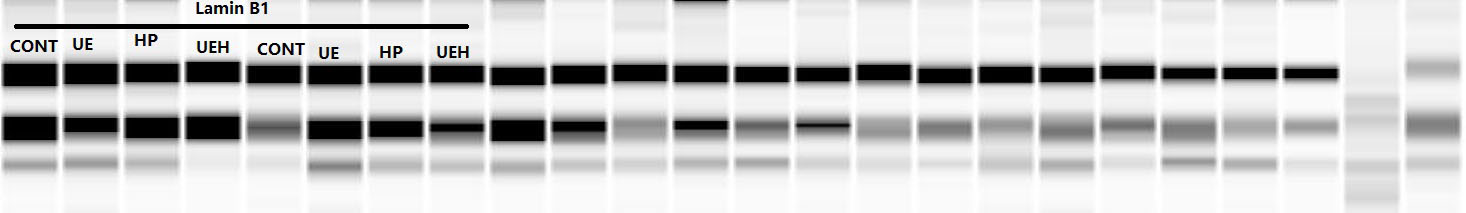


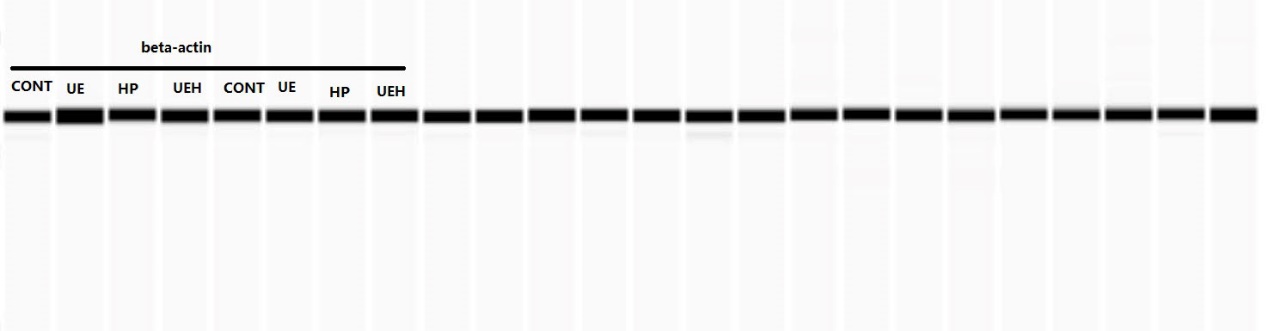


**Supplementary Figure 2 Microscopy images for results of Figure 5A.**

**CONT group**


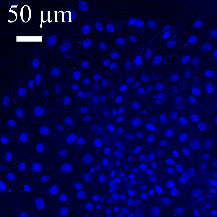

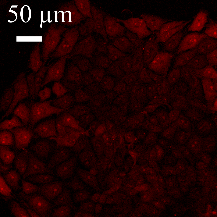

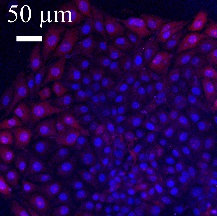


**DAPI for nucleus ROS Merged result**

**UE group**


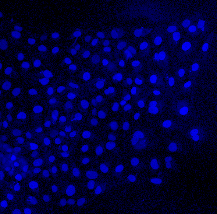

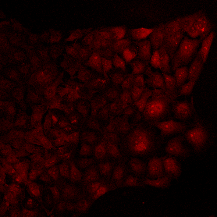

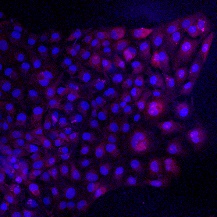


**DAPI for nucleus ROS Merged result**

**HP group**


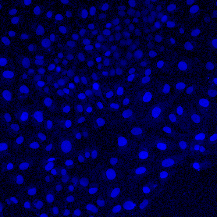

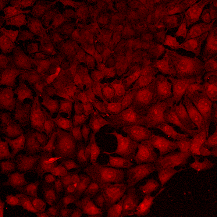

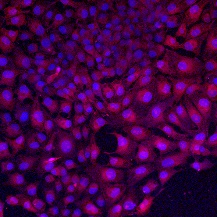


**DAPI for nucleus ROS Merged result**

**UEH group**


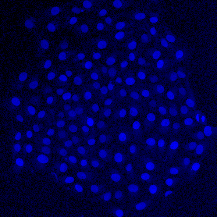

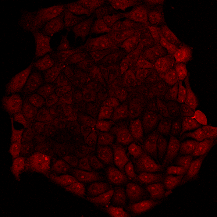

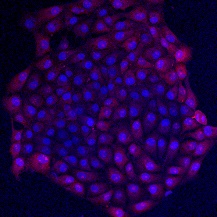


**DAPI for nucleus ROS Merged result**

**COMP group**


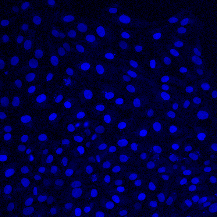

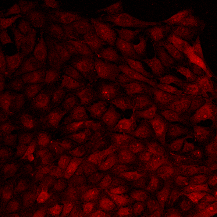

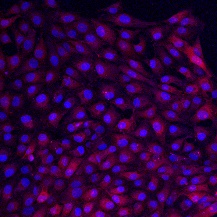


**DAPI for nucleus ROS Merged result**

**Supplementary Figure 3 Microscopy images for results of Figure 5B.**

**CONT group**


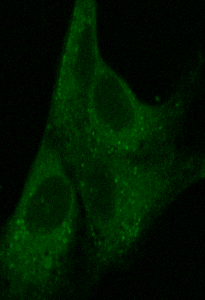

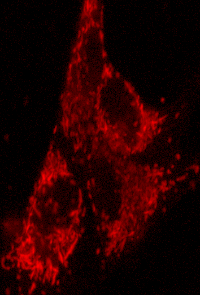

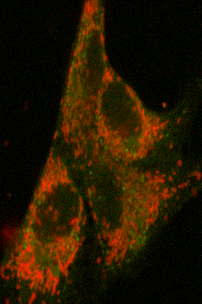


**JC-1 monomers (green) JC-1 aggregates (red) merged result**

**UE group**


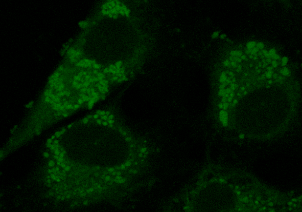

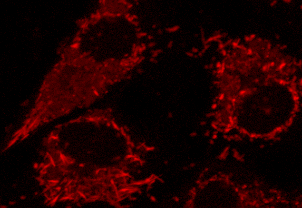

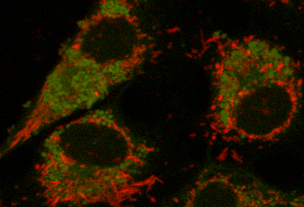


**JC-1 monomers (green) JC-1 aggregates (red) merged result**

**HP group**


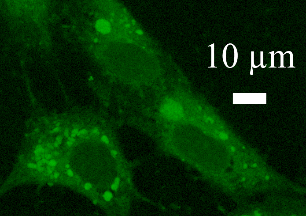

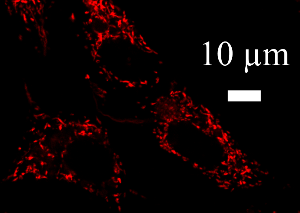

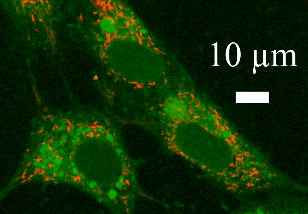


**JC-1 monomers (green) JC-1 aggregates (red) merged result**

**UEH group**


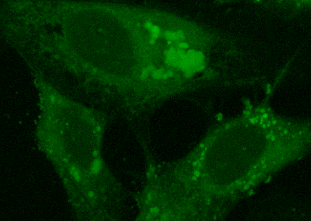

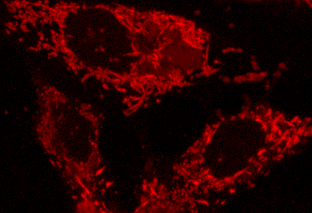

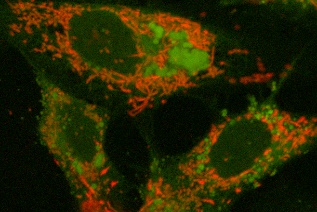


**JC-1 monomers (green) JC-1 aggregates (red) merged result**

**COMP**


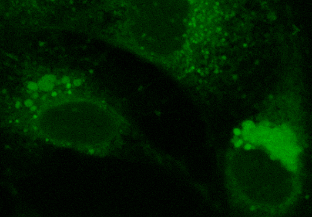

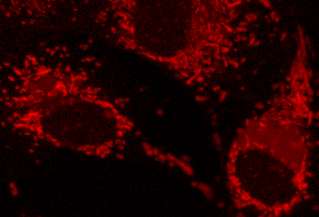

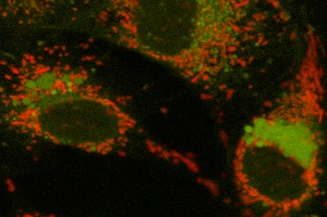


**JC-1 monomers (green) JC-1 aggregates (red) merged result**
